# Mediterranean Journal of Rheumatology December 2019 Highlights

**DOI:** 10.31138/mjr.30.4.194

**Published:** 2019-12-31

**Authors:** Theodoros Dimitroulas

**Affiliations:** Aristotle University of Thessaloniki, Thessaloniki, Greece

We welcome you to the December 2019 issue of the MJR. It contains a number of interesting contributions, from statistics in modern medical research to fasting diet and autoimmunity and vitamin-D in osteoarthritis. There are also several case reports covering various aspects of systemic diseases.

We live in an era where statistical analysis constitutes a crucial component of medical research. However, the statistical methods employed in submitted manuscripts do not always meet the high standards required. Importantly, most statistical errors could be avoided with appropriate training and sufficient planning. In this issue, Tsiamalou and Brotis^1^ present a practical guide – especially for young investigators – on the proper use of statistics in modern clinical research: they provide many useful tips aiming to avoid the most common statistical pitfalls. Addressing aspects of the same issue from a different perspective, Coskun Benlidayi^3^ reviews how statistics could and should be implemented in each and every step of research work, from hypothesis generation and testing to appropriate presentation and reporting of results.

In this issue we initiate the publication of articles submitted to the special theme: “Diet and Rheumatic Diseases” (Lead Editor Prof. Dimitrios Bogdanos). Intermittent fasting has emerged as a very popular eating pattern among people looking to lose weight. A comprehensive review by Venetsanopoulou and colleagues^2^ outlines the effects of fasting on autoimmune responses and how such diets can influence the activity of inflammatory arthritides.

Vitamin D has pleiotropic effects on several tissues: it has been associated with cartilage regeneration in arthritic joints although the exact mechanism remains to be defined. Vitamin-D deficiency has been linked with increased risk of developing osteoarthritis with some studies supporting and others demonstrating no evidence to support this hypothesis. A paper from Iran demonstrates a significant correlation between radiographic progression in plain x-rays and lower levels of Vitamin-D in individuals with knee osteoarthritis.^4^ The authors rightly state that more research is warranted in order to draw definite conclusions about this association.

A number of educational case reports describe the assessment and management of rare clinical problems, including central nervous system involvement in anti-synthetase syndrome treated with cyclophosphamide and mycophenolate mofetil,^5^ as well as IgG4-related disease manifested as a retroperitoneal mass in a patient with Behçet’s disease.^7^

Rauf et al.^6^ report an interesting case of giant cell hepatitis diagnosed in a 68-year-old male patient with clinical symptoms and serology encompassing a wide spectrum of autoimmunity such as Raynaud’s syndrome, interstitial lung disease, arthralgia, Jo-1 positivity as well as triple-positive antiphospholipid antibodies. This case underlines the significance of commonly underappreciated liver disease in systemic inflammatory diseases and suggests that treatment with mycophenolate mofetil may be effective for various guises of autoimmunity.

Implementation of tailored exercise programs for the management of inflammatory conditions and musculoskeletal problems in general remains an unmet need in daily clinical practice. One of the main reasons is lack of awareness from clinicians and allied health professionals of the overall benefits of such activities in chronic diseases. In that respect, Metsios and colleagues present an international collaboration involving seven countries aiming to develop e-learning courses to familiarize doctors and allied professionals with the value and favorable outcomes of exercise activity in patients with rheumatic diseases.^8^

We would like to take this opportunity to wish you a nice ending to 2019 and a great New Year 2020. Through the pages of MJR, we will continue to try to cover contemporary, important and interesting aspects of rheumatic diseases. We thank all of you, authors, reviewers, members of the editorial board, publishing team and invite you to continue contributing to the further development of the journal in the new year and beyond.
